# Hospital Acquired Bacteremia in Vulnerable Patient Populations: A Deeper Dive Into Risk and Treatment Considerations

**DOI:** 10.1017/ash.2025.348

**Published:** 2025-09-24

**Authors:** Samantha Bastow, Kalvin Yu

**Affiliations:** 1 Becton Dickinson and Company; 2 Becton, Dickinson & Co.; Molly Jung, ; ChinEn Ai, ; Diane Flayhart, Antimicrobial Resistance Fighter Coalition/BD

## Abstract

**Background:** Immunocompromised patients are at an increased risk of infections. With the introduction of the new Hospital Onset Bacteremia and Fungemia (HOB) quality metric, it remains uncertain whether the incidence would be higher within these vulnerable populations. Additionally, the rate of antimicrobial resistance (AMR), which complicates the infection management, is not completely understood. We aim to provide insights into the incidence of HOB in cancer, transplant and surgical patients as well as challenges posed by AMR. **Method:** Data from three multi-center retrospective studies are included: 1. Adult patients in 38 US hospitals between October 2015 and June 2019 with a procedure under the National Healthcare Safety Network (NHSN) surveillance for SSI to assess the incidence of SSI and SSI-HOB co-occurrence; 2. Adult patients in 41 US hospitals between October 2015 and June 2019 with DRG for myeloproliferative (MP) cancer, solid tumor cancer, transplant, and non-cancer/non-transplant (“reference group”) to quantify their association with HOB; 3. Evaluation of adult patients in 168 hospitals between April 2018 and December 2022 to assess the rate of AMR pathogens and the proportion of AMR among bacterial isolates in patients with and without cancer. **Results:** 1.Rate of hospital-reported SSI was 0.15 per 100 admissions and admissions with SSI had significantly higher incremental cost ($30,689) and length of stay (LOS) (11.6 days); further, the incidence of HOB was 6-fold higher in admissions with SSI and SSI admissions with HOB resulted in additional $24,586 to cost of care and 6.3 days to the LOS; 2. Rate of HOB in MP cancer was 2-7-fold higher and 57% to 4-fold higher in transplant patients compared to the reference group, depending on LOS. There were no statistically significant differences in the risk of HOB between solid tumor cancer and the reference; 3. AMR pathogen rates were higher in cancer patients than patients without cancer for most pathogen groups, including vancomycin-resistant enterococci (IRR 1.95), extended-spectrum beta-lactamase (ESBL) producers (IRR, 1.48), carbapenem-nonsusceptible Enterobacterales (IRR, 1.46) and multidrug-resistant Pseudomonas aeruginosa (IRR, 1.31). The percentage of nonsusceptible isolates in most pathogen groups was lower in patients with cancer versus without cancer except for ESBL producers among Enterobacterales and vancomycin resistance among enterococci, which were higher in cancer patients. **Conclusion:** Certain vulnerable patient populations were found to be at greater risk of HOB including those with SSI, MP cancer and transplant patients. The higher incidence of AMR in cancer patients further complicates management of high-risk infections.

